# Comprehensive evaluation of peptide *de novo* sequencing tools for monoclonal antibody assembly

**DOI:** 10.1093/bib/bbac542

**Published:** 2022-12-21

**Authors:** Denis Beslic, Georg Tscheuschner, Bernhard Y Renard, Michael G Weller, Thilo Muth

**Affiliations:** Robert Koch Institute, MF1, Nordufer 20, 13353 Berlin; Federal Institute for Materials Research and Testing (BAM), Richard-Willstätter-Straße 11, 12489 Berlin; Hasso Plattner Institute, Digital Engineering Faculty, University of Potsdam, Prof.-Dr.-Helmert-Straße 2-3, 14482 Potsdam; Federal Institute for Materials Research and Testing (BAM), Richard-Willstätter-Straße 11, 12489 Berlin; Federal Institute for Materials Research and Testing (BAM), Richard-Willstätter-Straße 11, 12489 Berlin

**Keywords:** *de novo* peptide sequencing, bioinformatics, benchmarking study, monoclonal antibodies, mass spectrometry

## Abstract

Monoclonal antibodies are biotechnologically produced proteins with various applications in research, therapeutics and diagnostics. Their ability to recognize and bind to specific molecule structures makes them essential research tools and therapeutic agents. Sequence information of antibodies is helpful for understanding antibody–antigen interactions and ensuring their affinity and specificity. *De novo* protein sequencing based on mass spectrometry is a valuable method to obtain the amino acid sequence of peptides and proteins without a priori knowledge. In this study, we evaluated six recently developed *de novo* peptide sequencing algorithms (Novor, pNovo 3, DeepNovo, SMSNet, PointNovo and Casanovo), which were not specifically designed for antibody data. We validated their ability to identify and assemble antibody sequences on three multi-enzymatic data sets. The deep learning-based tools Casanovo and PointNovo showed an increased peptide recall across different enzymes and data sets compared with spectrum-graph-based approaches. We evaluated different error types of *de novo* peptide sequencing tools and their performance for different numbers of missing cleavage sites, noisy spectra and peptides of various lengths. We achieved a sequence coverage of 97.69–99.53% on the light chains of three different antibody data sets using the de Bruijn assembler ALPS and the predictions from Casanovo. However, low sequence coverage and accuracy on the heavy chains demonstrate that complete *de novo* protein sequencing remains a challenging issue in proteomics that requires improved *de novo* error correction, alternative digestion strategies and hybrid approaches such as homology search to achieve high accuracy on long protein sequences.

## Introduction

Monoclonal antibodies (mAbs) are immunoglobulins of unique specificity generated artificially in laboratories to mimic antibodies produced by the immune system [1]. Their reproducibility under certain conditions and high binding affinity to target molecules make them essential to various diagnostic and analytical applications in immunology, clinical chemistry, food chemistry, environmental analysis, biochemistry, therapeutics and medicine [2–4]. Recently, multiple authors reported how antibodies lack proper classification and identification as research tools, thereby causing a so-called reproducibility crisis [5]. The results of multiple landmark papers could not be replicated because mAbs often lacked crucial quality control steps for correct characterization [6, 7]. One essential step for improving the research quality includes the confirmation of the amino acid sequence [8, 9]. In addition, retrieving sequence information of antibodies is crucial for understanding the structural basis of antibody–antigen binding, recognition and interaction [10]. The structural basis for the specificity in protein–protein interactions lies in the sequence diversity of antibodies. The majority of sequence diversity focuses on the hypervariable loops within the variable regions of antibodies, called complementarity-determining regions (CDRs), which are mainly responsible for the interaction between the antibody and their target structures [10, 11]. Most established methods for antibody *de novo* sequencing rely on sequencing mRNA from hybridoma cells. However, these approaches all depend on the availability of pure clones of antibody-producing cells [12]. Moreover, crucial posttranslational modifications, which affect antigen binding, developability and effector functions, cannot be detected by DNA sequencing [3]. Hence, approaches to sequence the antibody on protein level are necessary.

Tandem mass spectrometry (MS/MS) is a powerful method for retrieving the amino acid sequence of peptides. Typically, in standard shotgun proteomics, protein samples are digested with proteolytic enzymes into shorter peptides, which are more suitable for analysis by MS/MS [13]. To obtain sequential information from novel or unknown proteins, *de novo* peptide sequencing is commonly used, which identifies peptides directly from MS/MS spectra without relying on a sequence database [14]. Here, each amino acid is derived by computing mass differences of ions from a fragmented peptide. As the manual characterization of peptides using *de novo* sequencing can be very time consuming and challenging, a variety of algorithms have been developed to differentiate signal ion peaks from noise peaks to predict the correct peptide sequence [14–16]. Recent advances in deep learning (DL) have marked an important milestone for database-independent prediction of peptide sequences from MS/MS data [16]. The encoder–decoder architecture was designed to solve specific tasks in sequence-to-sequence learning [17]. Tran *et al.* [18] employed convolutional neural networks (CNNs) to encode mass spectra while using recurrent neural networks (RNNs) as a decoder to predict the amino acids of peptide sequences one by one. Their method DeepNovo outperformed state-of-the-art methods at that time. Multiple methods have been published based on the network architecture of DeepNovo, namely, DeepNovo-DIA [19], SMSNet [20] and PointNovo [21]. More recently, the transformer-based framework Casanovo showed promising results for the prediction of peptide sequences [22].

Although peptide *de novo* sequencing has improved in recent years, the full-length assembly of protein sequences poses another challenging task. In most cases, database search algorithms, such as MSGF+ [23], infer the correct proteins from identified peptide sequences [24]. However, the determination of protein sequences, which are not part of public databases, limits the feasibility of this approach. In the case of unknown antibodies, the variable sequence is not available and cannot be derived from database search algorithms [25]. Hence, *de novo* peptide sequencing and the assembly of the predicted peptides are necessary for assessing the amino acid sequence of unknown antibodies. Currently, only a few developed methods were reported for database-independent full-length antibody *de novo* sequencing and assembly, for instance, meta-SPS [26], ALPS [27], pTa [25] and MuCS [28]. Meta-SPS utilized overlapping fragment ion peaks from different spectra to construct meta-contigs before *de novo* sequencing. Across six diverse proteins and the aBTLA antibody, the authors observed a sequence coverage between 68 and 99%. Nonetheless, meta-SPS faces multiple limitations and is not combinable with recently developed *de novo* sequencing algorithms [26]. Tran *et al.* analyzed antibodies using PEAKS *de novo* [29], PEAKS DB [30] and the homology software SPIDER [31] in a complementary way. The results from these three algorithms serve as input for their de Bruijn assembler ALPS. Still, despite using homology and database search algorithms, the authors inspected a fragmented and incomplete assembly of long antibody sequences, particularly at the variable region of the heavy chain [27]. Thus, *de novo* sequencing of proteins remains a challenging and important problem to date.

Most publications regarding new *de novo* peptide sequencing approaches include a performance comparison of recently developed tools [14, 32, 33], yet, to our knowledge, there is no published independent evaluation of different *de novo* sequencing algorithms on antibody data sets. Moreover, newly developed *de novo* peptide sequencing tools rarely include antibodies for benchmarking their method in comparison to already existing tools [20–22]. Only the authors of DeepNovo used antibodies as an example application for their tool [18]. *De novo* sequencing studies involving antibody data mostly deal with validating antibody-specific assembly tools [25–27] or introducing alternative experimental methods [12, 34]. In this study, we present a performance evaluation of six recently developed *de novo* sequencing algorithms (Novor, pNovo 3, DeepNovo, SMSNet, PointNovo and Casanovo), which we chose based on their availability and performance in previous studies [14, 21, 22, 33]. In contrast to previous studies, we evaluate *de novo* peptide sequencing tools on various enzymatic antibody data sets. Furthermore, we investigate common error types, the impact of noisy spectra and missing fragmentation ions. To compare the ability of previously mentioned tools to reconstruct full-length protein sequences without additional database algorithms, we employed the de Bruijn assembler ALPS. Finally, we discuss possible solutions and the demanding challenges of *de novo* antibody sequencing.

## Materials and methods

### Antibody data sets

We analyzed data from three publicly available antibody data sets. The data sets were downloaded from the proteomics MS data repositories PRIDE [35] and MassIVE [36]. Table 1 gives an overview of the three evaluated antibody data sets, showing the reference, available digestion, mass instrument, ionization type and fragment ion resolution. The Glu-C file of IgG1-Human-LC and the trypsin file of WIgG1-Mouse-LC were corrupted and therefore not included in our analysis. In total, we analyzed 25 different MS/MS experiments (Supplementary Figure S1).

**Table 1:** Overview of evaluated antibody data sets. For each data set, we provided the name of the data set, the ID, the mass instrument, the ionization type, the fragment ion resolution in FWHM, a reference and the number of proteolytic enzymes in the data set

Data set name	Database ID	Mass instrument	Ionization type	Resolution	Ref.	Enzymes
IgG1-Human	MSV000079801	LTQ Orbitrap	HCD	17 500	[27]	Trypsin, chymotrypsin, asp-N, lys-C, glu-C, proteinase K
WIgG1-Mouse	MSV000079801	LTQ Orbitrap	HCD	17 500	[27]	Trypsin, asp-N, chymotrypsin
Herceptin	PXD023419	Orbitrap Fusion	Stepped HCD and EThcD	30 000	[12]	Trypsin, thermolysin, lys-N, lys-C, glu-C, asp-N, aLP, chymotrypsin, elastase

### Data processing

#### De novo sequencing methods

Table 2 provides an overview of the evaluated *de novo* peptide sequencing algorithms. Furthermore, it provides information about the algorithmic paradigm, the project website and the corresponding reference.

**Table 2:** Overview of all *de novo* sequencing tools used in this study. For each algorithm, the name of the tool, the algorithmic paradigm, the year of the publication, the reference and the project website of the corresponding method are displayed

Software	Algorithmic paradigm	Year	Ref.	Project website
Novor	Spectrum graph, machine learning, decision tree	2015	[37]	rapidnovor.com/
pNovo 3	Spectrum graph, machine learning, SVM	2019	[38]	i.pfind.org/
DeepNovo	DL, CNN + RNN	2017	[18]	github.com/nh2tran/DeepNovo/
SMSNet	DL, CNN + RNN	2019	[20]	github.com/cmb-chula/SMSNet/
PointNovo	DL, PointNet+RNN	2021	[21]	github.com/volpato30/PointNovo/
Casanovo	DL, transformer	2022	[22]	github.com/Noble-Lab/casanovo

##### Description of de novo sequencing algorithms used

Novor [37] is based on a decision tree scoring function to select peptide predictions. pNovo 3 [38] employs a learning-to-rank framework using gap features and predictions from pDeep [39] to improve the scoring of peptide sequences. Novor and pNovo 3 were developed based on the spectrum-graph approach while using extensive machine-learning algorithms for an enhanced scoring function. DeepNovo [18] was the first approach to incorporate the encoder–decoder paradigm for *de novo* peptide sequencing. SMSNet [20] uses a similar CNN- and RNN-based framework but additionally includes optional post-processing and a shift layer in the encoder module. PointNovo [21] adopts an order-invariant network structure for the prediction process of higher-resolution data. Casanovo [22] employs a transformer-based framework to process and predict sequences of amino acids instead of using RNNs.

##### Preprocessing

Each instrument vendor uses its own file formats to store results from MS/MS experiments. These raw files need to be converted to open-format files to be compatible with *de novo* sequencing tools [40]. We reformatted the raw MS/MS data files from the previously mentioned data sets to Mascot Generic Format (MGF) using ProteoWizard [41]. A MGF file stores the *m/z* and intensity pairs of multiple mass spectra in a single text format. *De novo* sequencing tools predict amino acid residues by accessing the mass differences between the MS/MS peaks [14].

##### Parameters for de novo sequencing algorithms

We executed Novor (v.1.05) via the DeNovoGUI command-line interface (v.1.16.6) [42]. We ran pNovo 3 (v.3.1.3) via its executable GUI, which included pre-trained models for specific enzymes. To perform a fair comparison between spectrum-graph-based tools like Novor and pNovo 3, which are released only with pre-trained models, and the DL algorithms, DeepNovo (v.PNAS), SMSNet, PointNovo (v.0.0.1) and Casanovo (v.3.0.0), we trained all DL-based tools on high-resolution MS/MS data from the human proteome using the HCD library from MassIVE, which consists of 1 114 503 different peptides [36]. Training them on specific antibody data would give DL programs an unfair advantage compared with pre-trained software. We split the spectra into training, validation and test sets at a ratio of 98:1:1 while making sure that the split data sets did not share any common peptides. Each model was trained for 10 epochs using pre-defined parameters from each tool. We executed all tools at a precursor tolerance of 10 ppm and fragment mass tolerance of 0.02 Da. For each algorithm, carbamidomethylation of cysteine (C + 57.02 Da) was set as a fixed modification. Oxidation of methionine (M + 15.99 Da) and deamidation of asparagine and glutamine (N + 0.98 Da and G + 0.98 Da) were set as variable modifications. DeNovoGUI and the DL tools were executed on a Linux server machine (100 cores, 64GB RAM). We executed pNovo 3 on a Windows 64-bit computer since the software was not supported by a Linux operating system.

#### Assembly of identified peptides

The predicted peptides were further processed by the de Bruijn sequence assembler ALPS [27] to evaluate the ability of different *de novo* sequencing tools to reconstruct complete protein sequences. As described by the authors, a k-mer size of 7 ensures a sufficiently high coverage of the amino acid sequence while preventing repetitiveness of the resulting contigs at the same time. ALPS takes the *de novo* confidence score into consideration for the assembly, but it could generate incorrect results using a high amount of low-confidence k-mers. The authors of DeepNovo recommend removing sequence contaminants from *de novo* sequencing results by excluding peptides with a confidence score below 50 to improve the quality of the assembly [18]. Since every single *de novo* sequencing algorithm calculates its confidence score in a different manner, we chose the threshold for the confidence score based on the amino acid-level precision. We removed peptide sequences below the confidence score of each tool for which the AA precision was below 50%. This aims to filter out low-quality predictions and, at the same time, ensures that we do not miss correctly predicted peptides, which have been assigned a low confidence score by the corresponding tool. We aligned the target contigs with the ground truth antibody sequence to classify the assembly results using BLAST [43]. Based on the alignments of the top contigs, we calculated the protein coverage and accuracy. The target sequence was regarded as being covered in case a contig was aligned to the target (sub)sequence. We calculated the accuracy by the number of correct sequence calls, which were aligned to the target sequence.

### Evaluation metrics

#### Database search

For validating *de novo* sequencing algorithms, we compared each prediction to a pseudo-ground truth, which is commonly obtained by database search [14,44]. Since the evaluated data sets do not include a labeled ground truth for each spectrum, we performed a database search using the antibody sequences as our protein database. We used the combined results of the database algorithms MS-GF+ [23] and X!Tandem [45], which were both executed via SearchGUI (v.4.1.7) [46] and post-processed via PeptideShaker (v.2.2.2) [47] on a 64-bit Windows computer. We filtered all resulting peptide-spectrum matches (PSMs) using a false discovery rate (FDR) of 1%. The combined results of two database algorithms and an FDR rate of 1% would generate a reliable pseudo-ground truth for the evaluation of the *de novo* sequencing tools. We chose the cleavage parameters according to the enzyme used in the provided input file. Furthermore, the search parameters included the same modifications and mass tolerance that we selected for the *de novo* sequencing algorithms.

#### Recall and precision

We compared the predictions of each *de novo* sequencing algorithm with the pseudo-ground truth peptides, which were identified by database search. Recall and accuracy were measured at the peptide and amino acid level. The performance at the amino acid level was measured by matching amino acids between the prediction and the ground truth. We applied the same evaluation metric adapted by DeepNovo, Novor and PointNovo [18,21,37]: amino acids were considered as matched ones if their masses were different by <0.1 Da and if the prefix masses before them were different by <0.5 Da.

#### Identification of fragment ions and noise

To evaluate the amount of noise and missing fragment ions in each spectrum, we labeled each peak as a peptide peak or a noise peak using the Pyteomics framework [48]. For each cleavage site, we tried to identify eight different ion types (b, b(2+), b-NH3, b-H2O, y, y(2+), y-NH3 and y-H2O) since all evaluated *de novo* sequencing algorithms take these ion types into consideration for retrieving the peptide sequence. If possible, we matched these ion types to corresponding peaks in the spectra within a tolerance of 0.5 Da. Otherwise, we declared the cleavage site as missing. We only considered noise peaks if their intensity exceeded the median noise intensity for each data set. The number of noise peaks above this threshold was used to calculate the noise factor, which is defined as the ratio of the number of high-intensity peaks and the number of fragment ion peaks. McDonnell *et al.* [33] applied this approach recently in their evaluation of *de novo* sequencing algorithms.

## Results

### Performance of *de novo* sequencing algorithms on antibody data at the peptide and amino acid level

We evaluated six state-of-the-art *de novo* peptide sequencing algorithms, namely, Novor, pNovo 3, DeepNovo, SMSNet, PointNovo and Casanovo. For this purpose, we used the antibody data sets described in section Antibody Data sets to measure the accuracy across different enzymes using metrics specified in section Evaluation Metrics. Using three antibody data sets, we relied on 183 873 MS/MS scans, from which 23 844 peptides were identified with database search. Peptide identifications by database tools served as ground truth for evaluating the predictions from *de novo* sequencing tools. By comparing *de novo* sequencing results to this reference, we were able to identify the number of correctly predicted amino acids and peptides for each tool.

**Figure 1:**
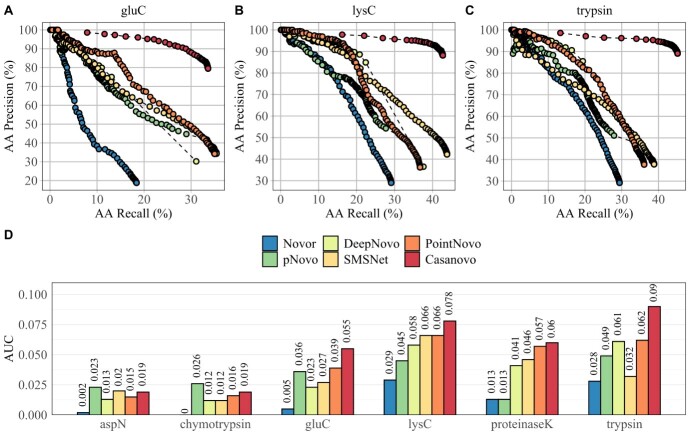
The PR curves of Novor, pNovo 3, DeepNovo, SMSNet, PointNovo and Casanovo for glu-C (A), lys-C (B) and trypsin (C) of the IgG1-Human-HC data set. The AUC of the six algorithms for each PR curve and each enzyme of IgG1-Human-HC (D).

Each algorithm generates a confidence score along the predicted sequence to reflect its quality. Setting a threshold to the confidence score outputs different sets of predicted peptides. A high threshold would show a small number of peptides with high precision, but it would exclude a large part of the data set, consequently reducing the recall. Here, we used different thresholds of the confidence score to draw precision-recall (PR) curves and used the area under the curve (AUC) as a summary metric for the accuracy of *de novo* sequencing results. Figure 1 displays the PR curves (A–C) and the AUC (D) of *de novo* sequencing tools across six different enzymes of the IgG1-Human-HC data set. Casanovo shows the highest AUC value across four enzymatic data sets because of its high AA precision compared with all other algorithms. All six evaluated algorithms display an overall higher AUC on trypsin and lys-C than on other proteases. The performance is generally lower on the enzymatic data sets of asp-N and chymotrypsin. The lower efficiency of non-tryptic enzymes for the prediction of peptide sequences was reported in different publications and had several reasons [49–51]. First, trypsin shows a higher number of PSMs, which is caused by a bias of database search algorithms toward peptides digested with trypsin [52]. Furthermore, peptides digested with trypsin are better suited for HCD fragmentation since they include at least one positive charge at each terminus, generating reliable b- and y-ion fragmentation patterns. In contrast, non-tryptic proteases may lack positive-charged termini, which makes it more challenging to identify the correct peptide [53]. The AUC on asp-N, chymotrypsin, glu-C and proteinase-K is considerably lower across all tools because of their distinct cleavage patterns. The general low AUC across all enzymes can be explained by the differences between the training data and the evaluated antibodies. The DL-based tools were not trained on antibody-specific data but on peptides from the human proteome data, which were derived from various experimental conditions [36]. Moreover, the evaluated data sets include a smaller number of unique peptides compared with available benchmarking data sets used in other studies [18, 21, 22]. The high AUC of pNovo 3 on non-tryptic peptides can be attributed to the pre-trained models for specific enzymes. A pre-trained enzyme-specific model was not available for proteinase-K, which explains the low accuracy of pNovo 3 on this data set.

**Figure 2:**
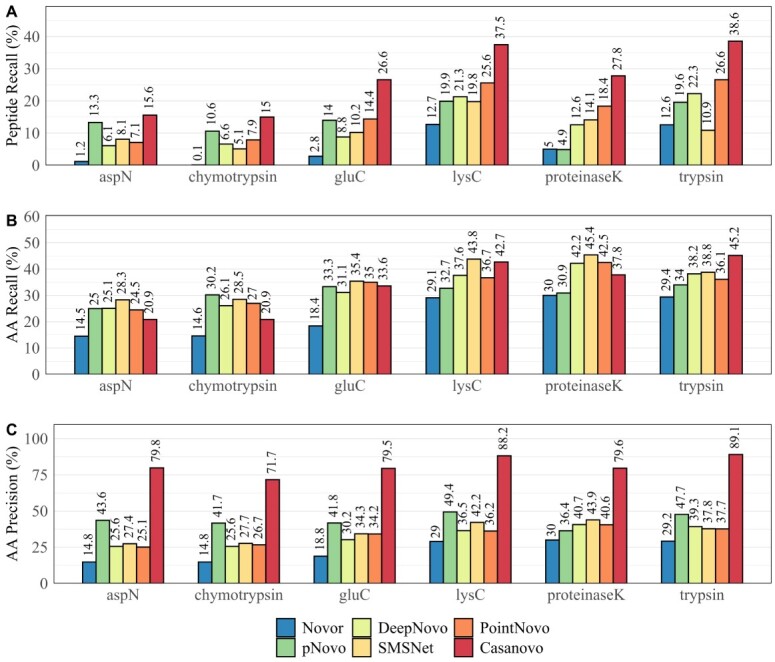
Total recall and precision of Novor, pNovo 3, DeepNovo, SMSNet, PointNovo and Casanovo across different enzymes on IgG1-Human-HC. (A) Recall at peptide level. (B) Recall at amino acid level. (C) Precision at amino acid level.

Figure 2 displays the total peptide recall (A), amino acid recall (B) and amino acid precision (C) across all six enzymatic cleavages of IgG1-Human-HC. In contrast to the results shown in Figure 1, we used all predictions from each tool regardless of their confidence score. Here, either SMSNet or PointNovo shows the highest amino acid recall in comparison to other tools on proteinase-K, asp-N, glu-C and lys-C (Figure 2B). Regarding the recall on peptide level, Casanovo exhibits the highest number of correct peptide predictions compared with all other *de novo* algorithms across all enzymes demonstrating the advantage of using transformers for peptide sequencing. Furthermore, Casanovo predicts amino acids with overall superior precision (Figure 2C). As Yilmaz *et al.* explained, the precursor *m/z* filter of Casanovo results in a prioritization of predicting full peptide sequences over partially correct subsequences [22]. Hence, Casanovo displays a very high AA precision and peptide recall, whereas its recall on amino acid level is comparable to PointNovo and SMSNet. The high accuracy of PointNovo can be attributed to its order-invariant networks, which have been applied for 3D recognition tasks and showed superior performance compared with state-of-the-art methods [54]. SMSNet profits from its shift layer, which helps to derive amino acids from MS/MS peaks [20]. Besides differences in the network structures, the number of fragment ions for predicting each amino acid residue plays another important role. While Novor and DeepNovo use eight ion types for predicting each position (y, y(2+), y-NH2, y-H2O, b, b(2+), b-NH2 and b-H2O), SMSNet and pNovo 3 take nine ion types into consideration for inferring peptides from spectrum peaks. Moreover, PointNovo examines 12 ion types to calculate theoretical *m/z* values at each prediction step. In contrast, the transformer-based framework of Casanovo processes the entire set of spectrum peaks at once. pNovo 3 predicted a similar or even higher number of correct peptides of the non-tryptic data sets asp-N and chymotrypsin than the DL tools because of its enzyme-specific models. Furthermore, pNovo 3 shows high accuracy on amino acid level compared with PointNovo, SMSNet and DeepNovo (Figure 2C) because of its extensive reranking process. Nonetheless, pNovo 3 cannot predict amino acids with the same precision as Casanovo.

### Evaluation of error types

**Figure 3:**
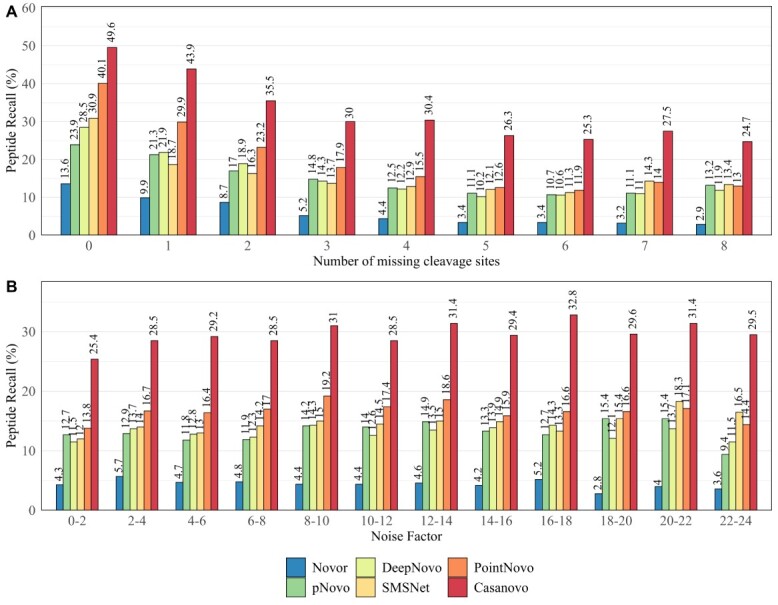
Total peptide recall of Novor, pNovo 3, DeepNovo, SMSNet, PointNovo and Casanovo across all data sets for different number of cleavage sites missing (A) and different noise factors (B) of the specific spectra.

Following the performance on peptide and amino acid level, we evaluated the source of incorrect predictions of the different *de novo* sequencing algorithms. McDonell *et al.* [33] reported previously that missing fragment ions and noise peaks pose a challenge for *de novo* sequencing algorithms. We observed that 90.51% of all 23 227 validated spectra were missing at least one fragment ion. Furthermore, we detected that 84.32% of all peaks from these spectra were classified as noise peaks. In Figure 3, we show the peptide recall for different numbers of missing cleavage sites and different noise factors of all validated spectra. As expected, *de novo* sequencing algorithms tend to identify a higher number of correct peptides from spectra with a lower amount of missing fragment ions (Figure 3A). Missing fragment ions decrease the overall performance of all *de novo* sequencing tools. Nonetheless, Casanovo displays a superior performance on spectra with up to eight missing cleavage sites. On spectra with at least four missing cleavage sites, all other DL-based tools show a low peptide recall of 9.56–14.62%, whereas Casanovo performs considerably better. Novor shows a noticeably lower performance compared with all other algorithms.

When viewed alone, the noise factor of different spectra does not have a strong effect on the accuracy of the *de novo* sequencing algorithms (Figure 3B). As McDonell stated, this is because of the stronger influence of the number of missing fragmentation sites on the peptide recall of each tool. Supplementary Figure S7 shows the impact of both the noise factor and the number of missed cleavages on the accuracy of pNovo 3, SMSNet, PointNovo and Casanovo. This demonstrates how a noise factor of at least 4 is already decreasing the prediction accuracy on spectra with no missing cleavage sites across all evaluated tools.

**Figure 4:**
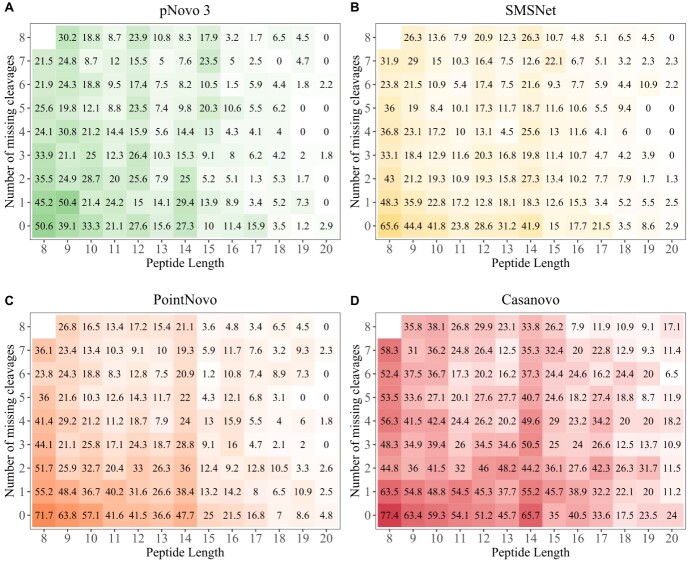
Heatmap showing peptide recall for different number of missing cleavages (*y*-axis) and peptide lengths (*x*-axis). Higher peptide recall is shown in green for pNovo 3 (A), yellow for SMSNet (B), orange for PointNovo (C) and red for Casanovo (D). Lower peptide recall is displayed in white. Spectra are not distributed uniformly and the squares on the right and top of the plots include fewer spectra, since combinations of long peptides and a high number of missing cleavages (top right) occur less likely.

Furthermore, we investigated the relationship between peptide length, the number of missing cleavage sites and prediction accuracy (Figure 4). The prediction accuracy decreases from short peptides with few missing fragmentation sites to long peptides with a high number of missing cleavages for each algorithm. The DL-based tools Casanovo, SMSNet and PointNovo show a higher prediction accuracy for peptides of a greater length compared with pNovo 3. PointNovo and SMSNet are able to learn sequence patterns of amino acids using their long short-term memory networks, which can overcome the issue of missing cleavage sites [18, 21]. The transformer-based approach allows Casanovo to process spectrum peaks as a whole and learn relationships between amino acids because of its self-attention mechanism. Spectrum graph-based methods show a lower peptide recall since missing fragmentation sites increase the complexity of possible peptide predictions [14]. McDonnell *et al*. [33] reported that the spectrum graph-based approach of Novor can correctly predict short subsequences of present fragment ions, in contrast to DeepNovo, which correctly predicts more complete peptides but fewer correct subsequences. However, even with all cleavage sites present, the evaluated algorithms only rarely identified correct peptides with a length of at least 18 amino acids. As expected, the number of correct predictions was higher for peptides below a size of 14 amino acids. Miscleavages lead to peptides of greater length, which would overall lower the prediction accuracy.

Following the influence of peptide length on the predictive performance, we compared the frequency of certain error types across multiple tools. We categorized incorrect peptide sequence predictions into 11 different error types and compared their relative amount between pNovo 3, SMSNet, PointNovo and Casanovo across all data sets (Table 3). We observed that for pNovo 3, SMSNet and PointNovo most errors were caused because of more than six wrongly assigned amino acids. Among the error types under 6 AAs, the inversion of the last 3 amino acids (PointNovo) and the replacement of 1 AA by 1 or 2 AAs (SMSNet, pNovo 3) appear as the most frequent origins of incorrect peptide predictions. Conversely, Casanovo generated fewer predictions with a smaller number of mismatches in relation to pNovo 3, SMSNet and PointNovo. Again, this can be attributed to Casanovo’s precursor filter, which results in a smaller fraction of errors, where more than 6 AAs were incorrectly predicted. The number of inversions was slightly lower on pNovo 3, demonstrating the advantage of a re-ranking framework for improved accuracy.

**Table 3:** Error types made by *de novo* sequencing algorithms tools pNovo 3, SMSNet, PointNovo and Casanovo on the data sets of IgG1-Human, WIgG1-Mouse and Herceptin. Shown are the total number of predictions, total number of errors and the relative amount of 11 different error types for each algorithm. ‘Other’ includes errors that do not fall into any other categories, e.g. ‘2 AAs replaced by 4 AAs’

Type of sequencing error	pNovo 3	SMSNet	PointNovo	Casanovo
Number of total predictions	16 170	23 227	22 417	10 907
Number of total errors	14 860	20 554	19 240	5118
Inversion first 3 AAs (%)	5.5	5.3	5.0	11
Inversion last 3 AAs (%)	2.4	4.4	6.7	14
Inversion first and last 3 AAs (%)	0.2	0.4	1.0	0.8
1 AA replaced by 1 AA or 2 AAs (%)	10	5.4	5.6	27
2 AAs replaced by 2 AAs (%)	7.0	4.5	4.2	9.4
3 AAs replaced by 3 AAs (%)	6.1	3.7	3.6	6.1
4 AAs replaced by 4 AAs (%)	2.5	3.2	2.8	4.8
5 AAs replaced by 5 AAs (%)	2.6	2.6	2.1	2.3
6 AAs replaced by 6 AAs (%)	3.1	2.3	2.2	2.1
More than 6 AAs wrong (%)	44	57	55	10
Other (%)	14	10	10	9.9

### Database-independent assembly of predicted peptide sequences

To validate the predictions of different peptide *de novo* sequencing tools on assembly level, we used the de Bruijn graph assembler ALPS, which generates several contiguous sequences (contigs) based on the *de novo* peptide results and their confidence scores [27]. We compared the longest constructed contig, the overall sequence coverage and the sequence accuracy for three antibody samples. As described in section Assembly of identified peptides, we considered only aligned contigs for the calculation of sequence accuracy. The light chains are 210–219 AAs long, whereas the heavy chains of our evaluated antibodies include over 440 AAs, which present a challenge for a complete sequence assembly. The longest constructed contig for the heavy chain of WIgG1 was generated by Casanovo, covering only 83 AAs (18.82%) of the protein sequence. On the light chain of WIgG1, the results of Casanovo were concatenated to a contig, which covered 110 AAs (50.23%) of the entire sequence.

Since single contigs only cover a small region of the full-length protein, we evaluated the protein sequence coverage using a higher number of contigs for the light chain of IgG1-Human (Table 4). Here, we only evaluated SMSNet, PointNovo and Casanovo, since these three tools showed a higher AA recall and peptide recall across various enzymes and data sets compared with DeepNovo, Novor and pNovo 3. Combined with Casanovo, we were able to assemble 97.69% (IgG1) to 99.53% (Herceptin) of the whole antibody sequence with an accuracy of 94.47–95.26%. We observed a similar high sequence coverage for SMSNet (90.74–97.20%) and PointNovo (93.15–99.07%). Interestingly, we were able to achieve high coverage and accuracy on the light chain of WIgG1, although we only used the enzymatic data sets of chymotrypsin and asp-N.

**Table 4:** Summary of *de novo* assembly results on light chains of three antibody data sets using the *de novo* peptide sequencing tools SMSNet, PointNovo, Casanovo, and the de Bruijn assembler ALPS (*k* = 7). We used the Top 20 contigs to compare the length, coverage and accuracy of mapped contigs. Mapped contigs must be aligned to the reference protein sequence. The longest contig describes the maximum length of all generated contigs. Sequence coverage was calculated as the percentage of amino acids of the complete protein sequence that was covered by at least one contig. Accuracy was calculated as the percentage of all protein sequence calls that were labeled correctly

	IgG1 LC (216 AA)	WIgG1 LC (219 AA)	Herceptin LC (214 AA)
SMSNet			
Mapped contigs	10	5	8
Longest contig	51 (23.61%)	61 (27.86%)	67 (31.30%)
Sequence coverage	196 (90.74%)	200 (91.32%)	208 (97.20%)
Sequence accuracy	171 (87.24%)	190 (95.00%)	183 (87.98%)
PointNovo			
Mapped contigs	7	3	6
Longest contig	51 (23.61%)	108 (49.32%)	75 (35.05%)
Sequence coverage	205 (94.91%)	204 (93.15%)	212 (99.07%)
Sequence accuracy	187 (91.22%)	191 (93.63%)	190 (89.62%)
Casanovo			
Mapped congis	7	4	4
Longest contig (AA)	65 (30.09%)	110 (50.23%)	105 (49.07%)
Sequence coverage (%)	211 (97.69%)	217 (99.09%)	213 (99.53%)
Sequence accuracy (%)	201 (95.26%)	205 (94.47%)	202 (94.84%)

Furthermore, we evaluated the assembly method on the heavy chains of our evaluated data sets (Supplementary Table S3). Here, we observed a lower sequence coverage and accuracy across all tools. Using Casanovo and ALPS, we achieved a sequence coverage of 76.39% (Herceptin) up to 93.72% (IgG1). We encountered multiple short overlapping contigs, which would make a full-length assembly more difficult without using additional tools. Moreover, these contigs include multiple mismatches, gaps, and were only partly aligned to the target sequence. Still, on the light chain of Herceptin, we achieved a sequence coverage of 99.53% using Casanovo.

Despite the challenges of full *de novo* protein sequencing, we were able to correctly assemble functionally important subregions, namely the variable region and the CDRs, with the use of Casanovo and ALPS (Supplementary Table S5). We identified the corresponding CDRs for each antibody using the Natural Antibody database [55]. The CDRs were 100% correctly predicted on the light chain of IgG1. The heavy chain was correctly assembled except for a single misidentification on CDR3. The incorrect sequence assignment included mismatches between amino acids with an identical mass (e.g. Q & GA; deamidated N & D; deamidated Q & E). The results on the variable regions and CDRs highlight the potential of DL-based *de novo* sequencing to identify unique antibody sequences.

## Discussion

In this study, we reviewed state-of-the-art *de novo* sequencing algorithms and applied them to the assembly of mAbs. We compared the performance of six recently developed and commonly used *de novo* peptide sequencing tools, namely, Novor, pNovo 3, DeepNovo, SMSNet, PointNovo and Casanovo.

Statistical analysis on amino acid and peptide levels revealed that the recently developed tools SMSNet, PointNovo and Casanovo achieved a high peptide recall on different enzymatic data sets (Figures 1 and 2; Supplementary Figures S2–S4). Similar to previous observations [21,56], DL-based algorithms predict a higher amount of correct peptide sequences compared with conventional spectrum-graph-based methods. A crucial factor for retrieving the correct peptide sequence is the resolution of the mass instrument and, consequently, the ability of *de novo* sequencing tools to make use of such high-resolution spectra [21]. For example, ambiguities between amino acids with similar mass (e.g. Q & K; oxidized M & F; AG & Q) cannot be resolved correctly on mass spectra with a wide fragment ion error tolerance of 0.1 Da and this makes MS/MS of higher resolution necessary [57]. As Qiao *et al.* pointed out, high-resolution spectra led to increased computational complexity for analyzing MS/MS data with *de novo* sequencing algorithms. DeepNovo and SMSNet need to discretize spectra with a higher resolution parameter, which increases the computation and memory demand, whereas PointNovo and Casanovo can handle high-resolution spectra without increasing their computational complexity [21]. However, particular amino acids cannot be resolved even with spectra and tools with higher resolution (e.g. I & L; Q & AG; deamidated N & D). Here, additional methods are necessary to retrieve the correct amino acid sequence. Discrimination of the isomeric residues isoleucine and leucine cannot be achieved via MS/MS but require MS3 fragmentation [58,59].

Cross-enzyme performance is an important quality feature of *de novo* sequencing methods in bottom-up proteomics. Yet, most publications regarding *de novo* sequencing tools rarely address the predictive abilities of non-tryptic proteolytic enzymes and focus on tryptic data sets because of their wide availability and well-established usage [19,38]. Qiao *et al.* [21] observed that enzyme-specific models had a notable influence on the performance and recommended training a separate model for each enzyme. However, training different models for over six enzymes can be a demanding task, especially if the data are only partly available or come from various sources with unequal experimental setups. Although our training data included mainly tryptic peptides, a relatively high number of non-tryptic peptides were identified by Casanovo, PointNovo, SMSNet and DeepNovo (Figure 2 and Supplementary Figure S3). Karunratanakul *et al.* [20] made a similar observation, where SMSNet was able to discover a large number of non-tryptic HLA-antigens, whereas 95% of its training data consisted of tryptic peptides. We conclude that DL tools can still be applied to different enzymatic data sets, although the performance will vary based on the cleavage pattern of the trained data set. In our opinion, the deployment of a higher number of enzyme-specific data sets and models would be beneficial for successfully applying *de novo* sequencing in proteomics. Furthermore, multienzyme DL models show the potential to improve the assembly of protein sequences [60].

Previous evaluations of *de novo* sequencing tools have observed an increased accuracy of these algorithms on simulated MS/MS spectra compared with real data sets [14, 33], suggesting that the bottleneck for *de novo* peptide identification lies in the quality of the provided data. As shown in our analysis, all evaluated tools show a higher peptide recall on spectra with fewer missing fragment ions (Figure 4). We observed that 90.51% of all spectra lacked at least one fragment ion. While newly developed tools demonstrate the potential of *de novo* sequencing, advanced post-processing steps are necessary to improve their accuracy. The DL-based tools SMSNet and PointNovo generated a higher number of completely incorrect peptides in comparison to pNovo 3 (Table 3). As Yang *et al*. [38] pointed out, DL models are directly learned from the MS/MS data and do not rely on well-designed features, which could help reduce the error frequency. Furthermore, the authors reported that even DL-based approaches have difficulties in distinguishing similar peptides with long-gapped subsequences, concluding that the quality of MS/MS data is a bottleneck of successful peptide prediction. However, the DL-based tool Casanovo displayed a lower number of incorrect predictions compared with pNovo 3, SMSNet and PointNovo, demonstrating the advantages of transformer-based models for predicting peptide sequences. The authors of Casanovo showed how a simple precursor mass filter yields much higher precision [22]. Moreover, it is worth mentioning that several methods were published, discussing how to improve the encoder–decoder paradigm of DL tools in proteomics [56,61]. Fei pointed out that deep neural networks face difficulties on tandem mass spectra with incomplete fragment patterns. Multiple authors have confirmed that a considerable amount of *de novo* sequencing errors occurs at the N-terminal ends because of the absence and low intensity of fragment ions [62,63]. Hence, Fei developed a retrieve-and-revise framework to compensate for low-quality spectra. His peptide identification model, which relies on a reference database, was able to outperform current state-of-the-art algorithms [56]. Ge *et al.* proposed the use of deep residual shrinkage networks for their *de novo* sequencing method DePS to improve the accuracy on noisy spectra with missing fragmentation ions. Their implementation improved the extraction of features from MS/MS spectra and outperformed DeepNovoV2 on multiple data sets [64]. Liu *et al.* [65] used multiple temporal convolutional network blocks to improve the current state of de novo sequencing with their tools PepNet. Recently, the transformer-based approach DPST [66] showed an increased accuracy in predicting peptide sequences compared with DeepNovo, while reducing the model complexity and inference time.

Despite the ongoing effort and progress in *de novo* peptide sequencing, reliable protein assembly is still a demanding task. Our findings show that the ability of database-independent approaches of full-length protein assembly is limited even when using multiple contigs and different *de novo* sequencing tools (Table 4). The longest generated contig only covers at best 21.82% of the heavy chain and 50.23% of the light chain. Using Casanovo and ALPS, we accomplished a sequence coverage of 97.69–99.53% on the light chains of our evaluated antibodies. However, additional database tools or homology algorithms are necessary to correctly assemble multiple short contigs to complete antibody sequences. Homology search can be combined together with *de novo* sequencing to improve the discovery of protein sequence information and overcome problems caused by mass segment errors [26,67,68]. Commercial software packages such as PEAKS AB [27] and Supernovo [11] use antibody germline sequences as a starting point together with *de novo* sequencing results to identify mAbs. Supernovo employs *de novo* peptide sequencing, database search, *in silico* genetic recombination and a final sequence assembly for an automatic antibody sequence prediction. Similarly, the publicly available software tool Stitch maps short peptide reads to user-defined templates for reconstructing monoclonal and polyclonal antibody sequences [69]. In addition to the before-mentioned homology tools, antibody-specific language models, such as AbLang, can help to restore missing residues of full protein sequences caused by sequencing errors without using a germline template sequence [70]. Thus, the development of publicly available frameworks and pipelines for automated assembly of *de novo* peptide sequencing results from recently developed algorithms would improve the reliable usability of *de novo* sequencing for full antibody assembly.

**Key Points**

- A comprehensive review of *de novo* sequencing tools in proteomics is provided that aims to solve the challenge of antibody sequencing and subsequent assembly.
- Improved sensitivity of deep learning-based tools was found in comparison to classical *de novo* sequencing algorithms, such as spectrum graph-based algorithms, across various enzymatic data sets of antibodies.
- The number of missing fragmentation sites, noisy spectra and long peptide sequences poses a limit for all *de novo* sequencing tools.
- Database-independent assembly of light chains can be achieved up to a sequence coverage of 99.53% by using the de Bruijn assembler ALPS together with *de novo* peptide predictions from Casanovo.
- Further development of freely available and automatized pipelines for an accurate assembly of peptide predictions is necessary to successfully retrieve full antibody sequences.

## Supplementary Material

SupplementaryMaterial_bbac542

## Data Availability

Result files and Python code to reproduce the results in this study are available at Figshare (doi.org/10.6084/m9.figshare.21394143).

## Notes

**Denis Beslic** is a doctoral candidate at the Robert Koch Institute in Berlin, Germany.

**Georg Tscheuschner** is a doctoral candidate at the Federal Institute for Materials Research and Testing (BAM) in Berlin, Germany.

**Bernhard Y. Renard** is professor for data analytics and computational statistics at Hasso-Plattner-Institute and the University of Potsdam in Potsdam, Germany.

**Michael G. Weller** is the head of section 1.5 (Protein Analysis) at the Federal Institute for Materials Research and Testing (BAM) in Berlin, Germany.

**Thilo Muth** is the head of section S.3 (eScience) at the Federal Institute for Materials Research and Testing (BAM) in Berlin, Germany.
