# Supplementary material for: Comprehensive evaluation of peptide de novo sequencing tools for monoclonal antibody assembly
Source: Brief Bioinform. 2022 Dec 21;24(1):bbac542. doi: 10.1093/bib/bbac542 (PMC9851299; doi:10.1093/bib/bbac542)
Supplement: SupplementaryMaterial_bbac542 [file supplementarymaterial_bbac542.docx]

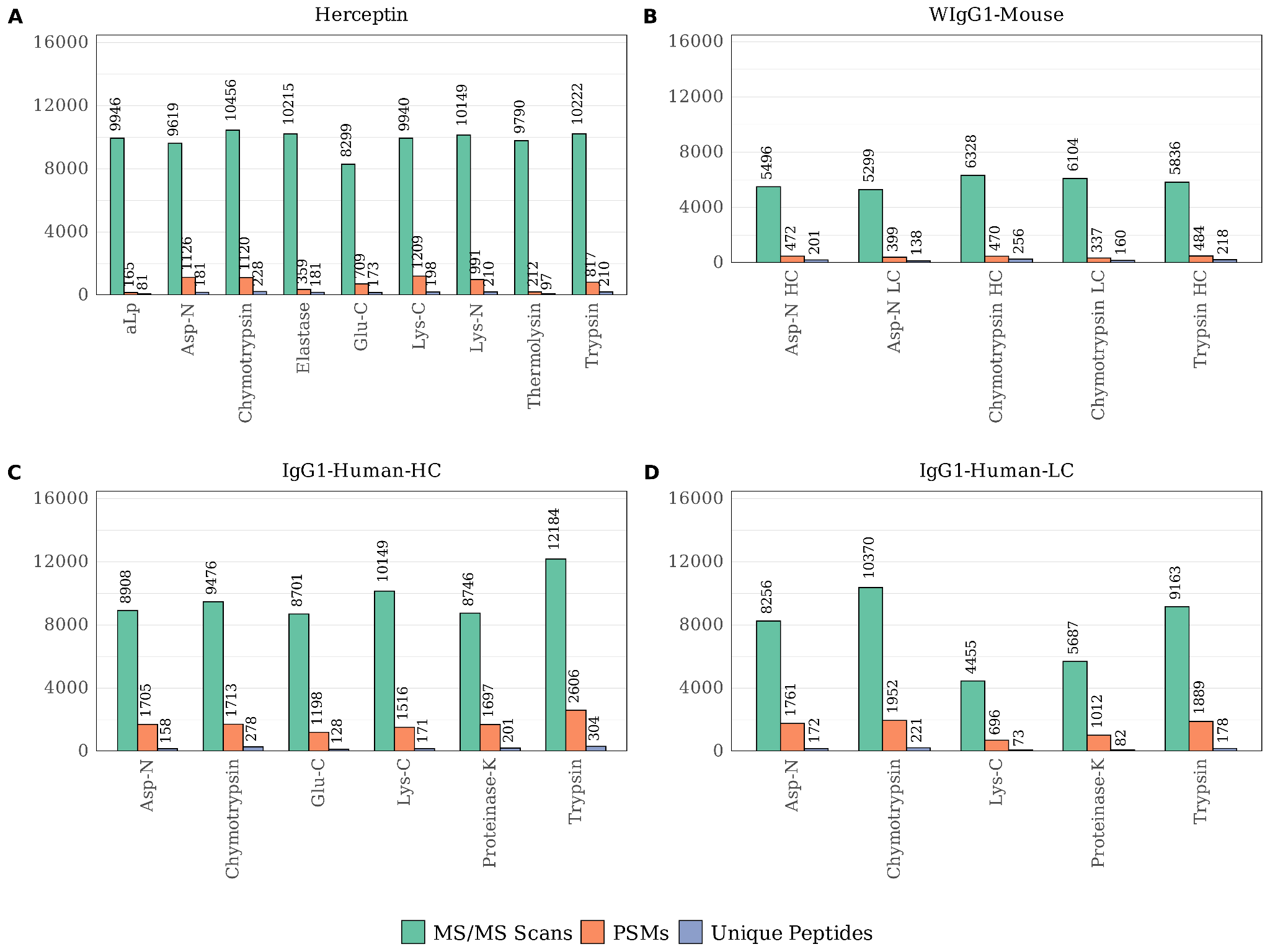


**Supplementary Figure S1** | Scan statistics for each protease digest of Herceptin (A), WIgG1-Mouse (B), the heavy chain (C), and the light chain (D) of IgG1-Human. For each dataset, the number of MS/MS scans (green), the number of peptide-spectrum matches (orange), and the number of unique peptides (blue), are displayed.


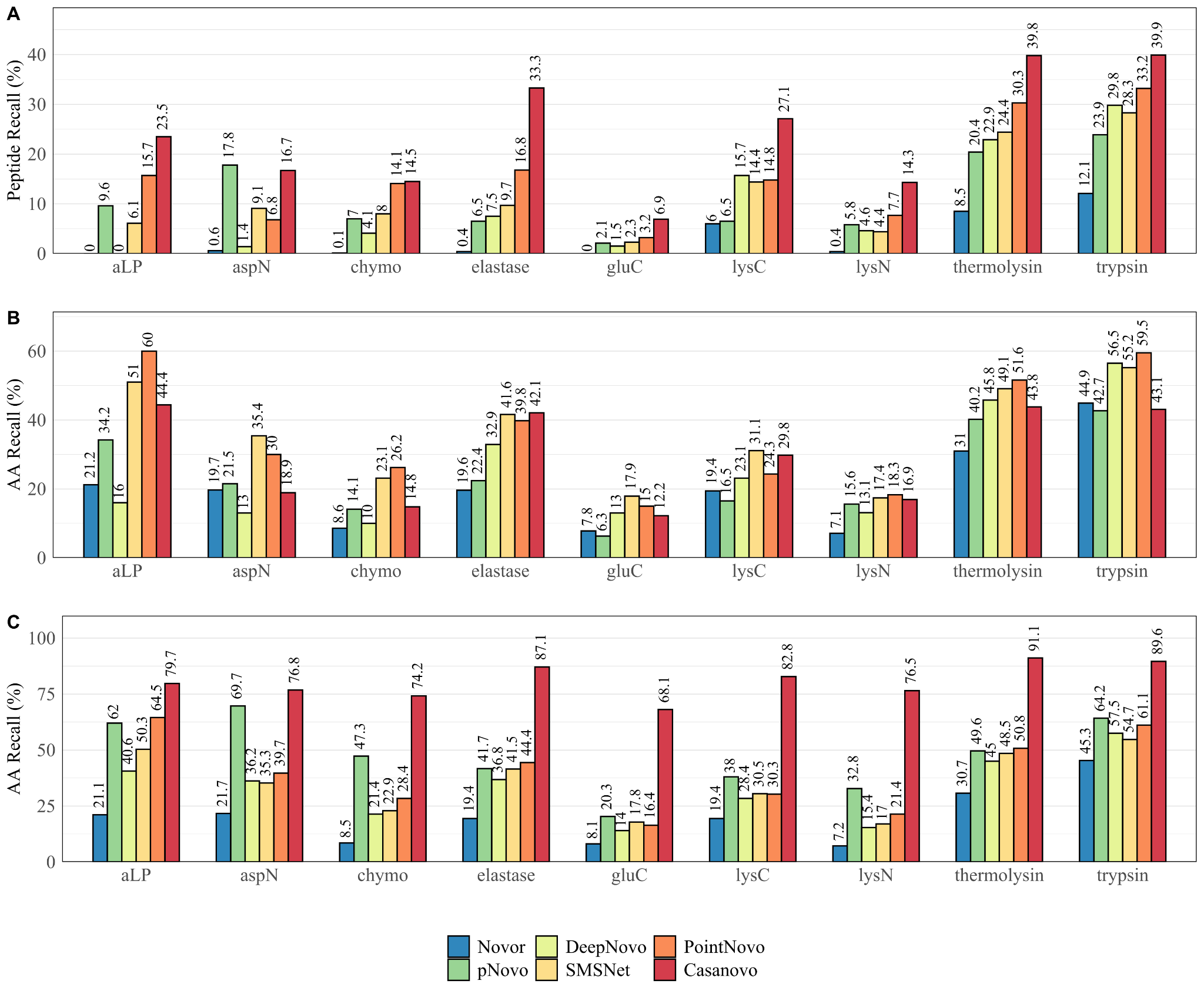


**Supplementary Figure S2** | Total recall and precision of Novor, pNovo 3, DeepNovo, SMSNet and PointNovo across different enzymes on Herceptin. (A) Recall at peptide level. (B) Recall at amino acid level. (C) Precision at amino acid level.


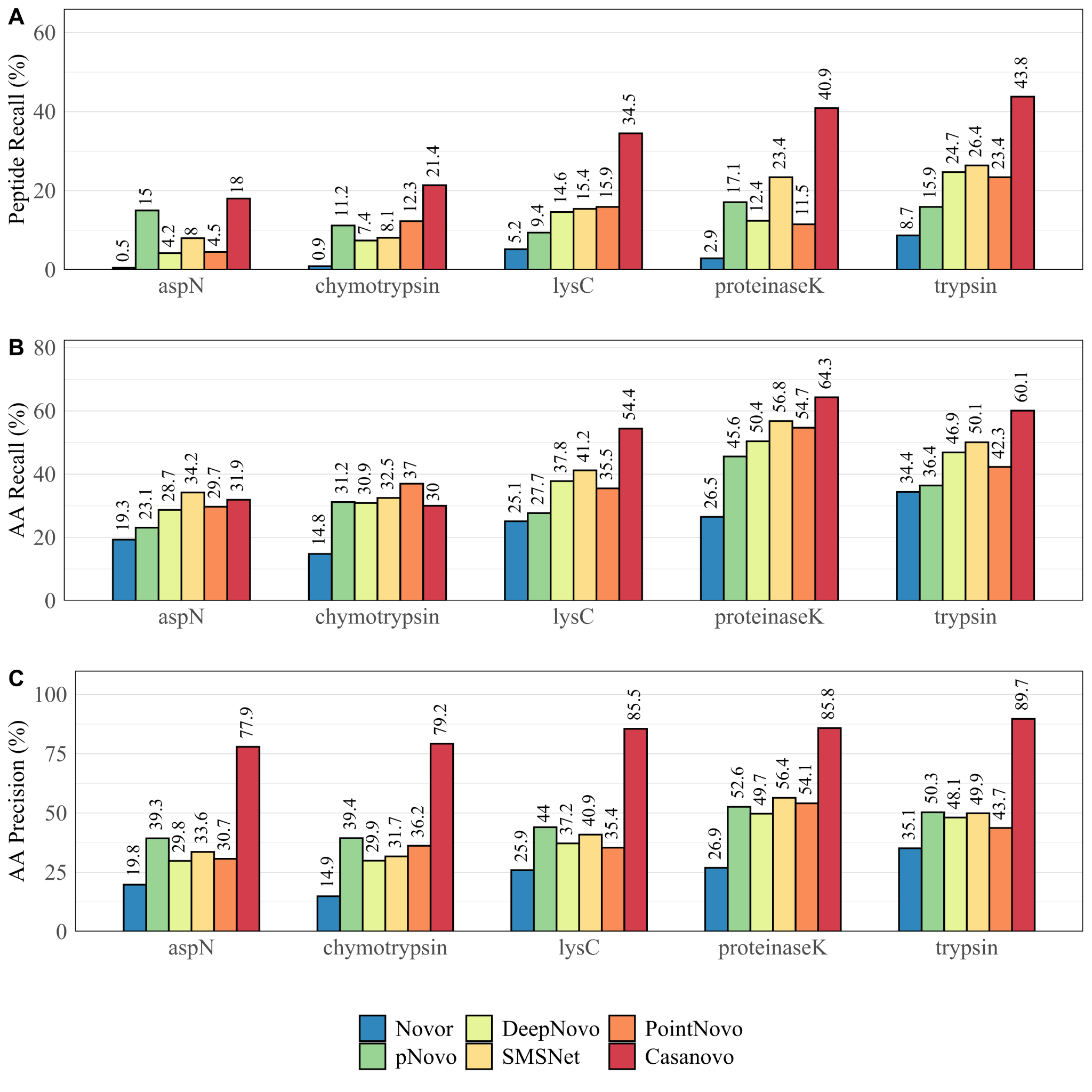


**Supplementary Figure S3** | Total recall and precision of Novor, pNovo 3, DeepNovo, SMSNet and PointNovo across different enzymes on IgG1-Human-LC. (A) Recall at peptide level. (B) Recall at amino acid level. (C) Precision at amino acid level.


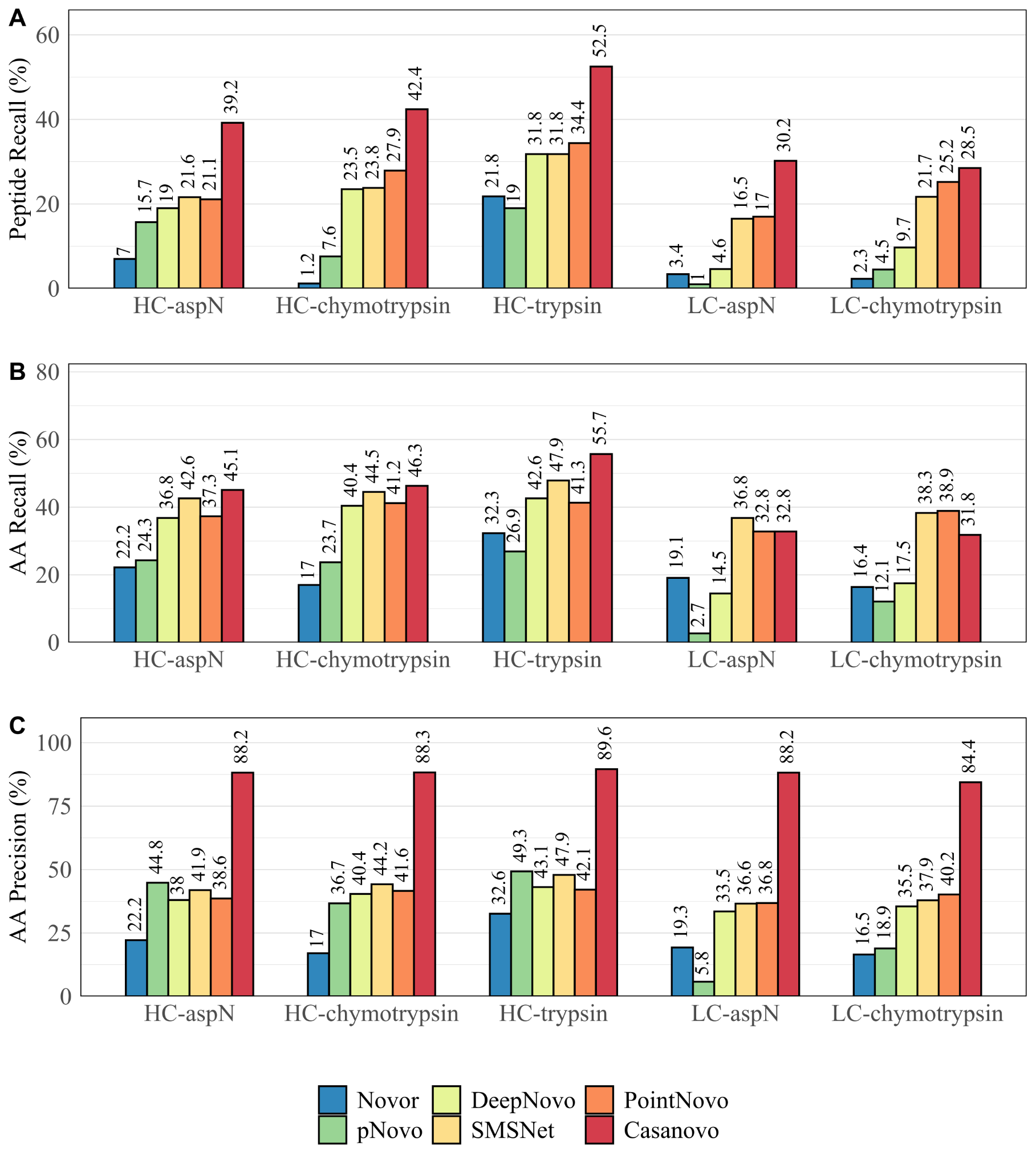


**Supplementary Figure S4** | Total recall and precision of Novor, pNovo 3, DeepNovo, SMSNet and PointNovo across different enzymes on WIgG1-Mouse. (A) Recall at peptide level. (B) Recall at amino acid level. (C) Precision at amino acid level.


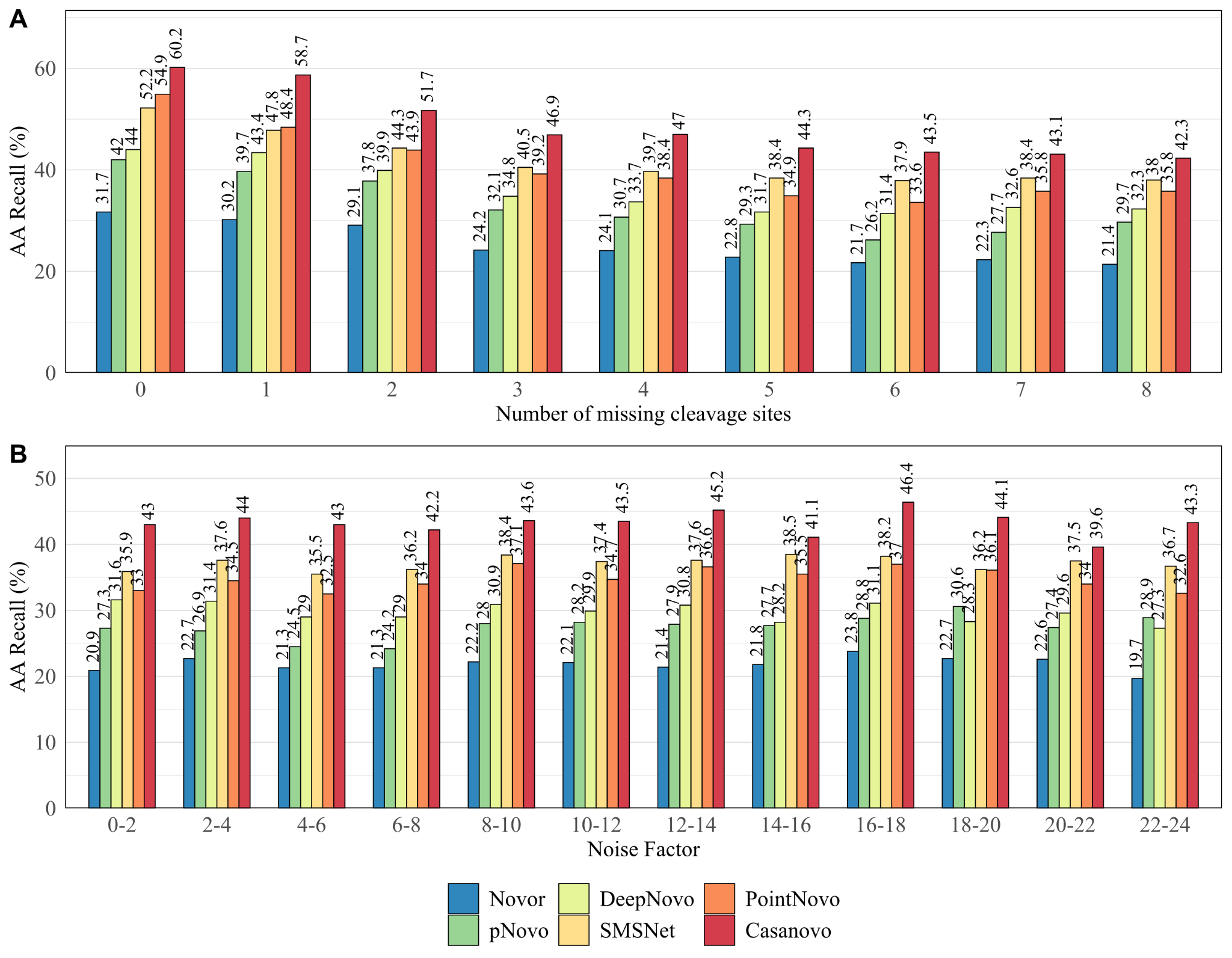


**Supplementary Figure S5** | Total amino acid recall of Novor, pNovo 3, DeepNovo, SMSNet, PointNovo, and Casanovo across all datasets for different number of cleavage sites missing (A) and different noise factors (B) of the specific spectra.


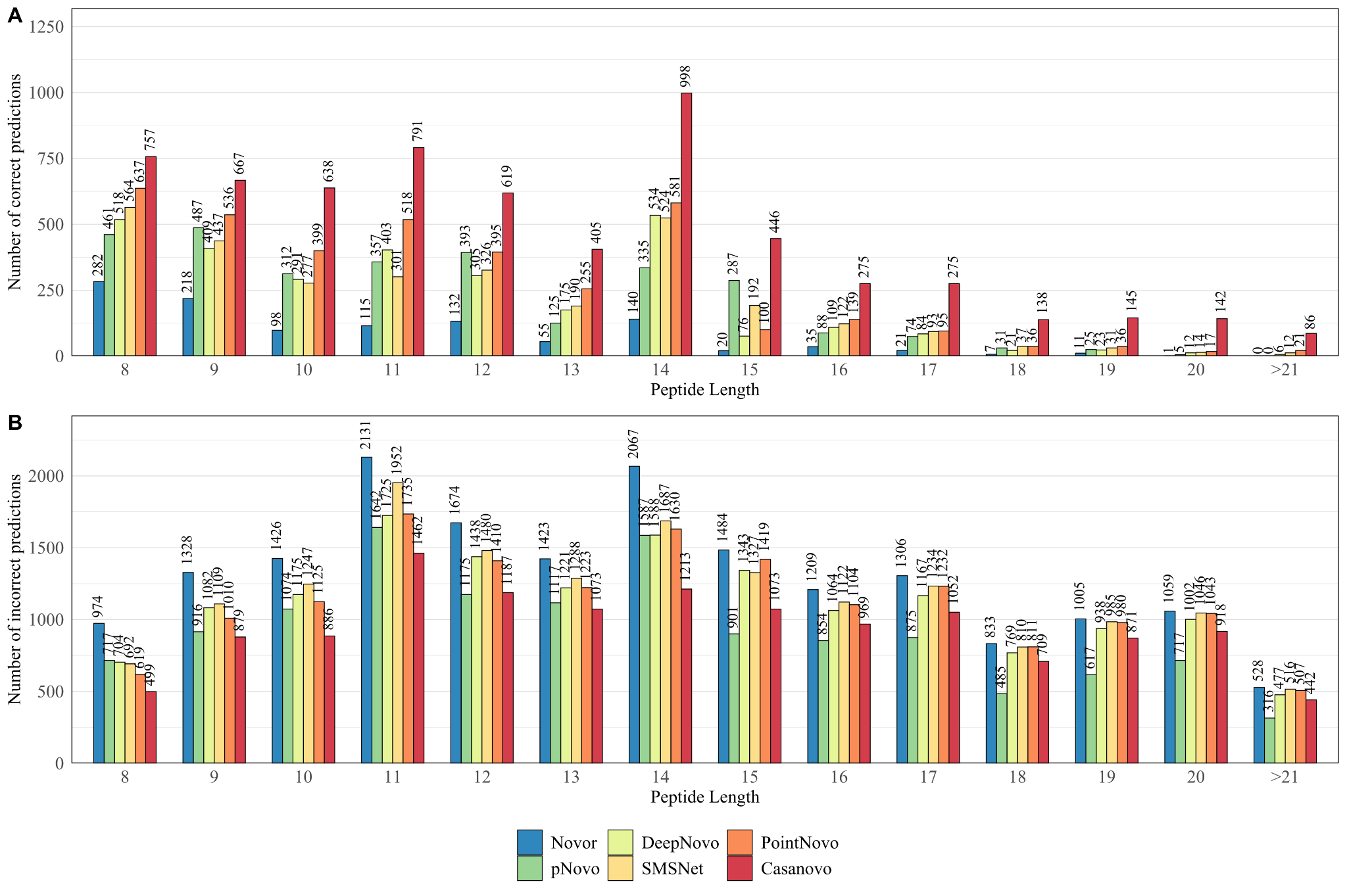


**Supplementary Figure S6** | Peptide length distribution of correct (A) and incorrect (B) predictions of Novor, pNovo 3, DeepNovo, SMSNet, PointNovo, and Casanovo on all datasets.


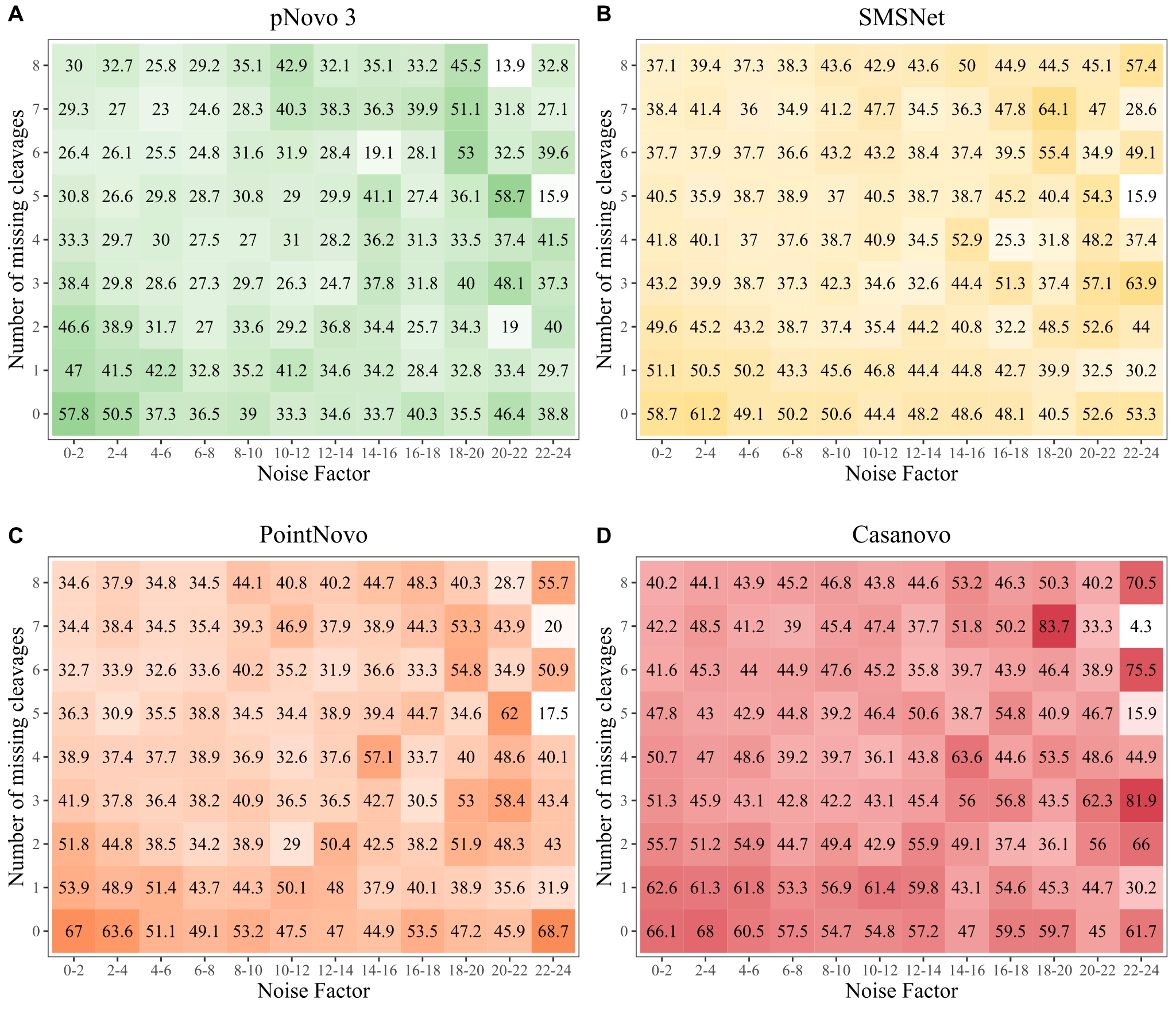


**Supplementary Figure S7** | Heat plot showing amino acid recall for different number of missing cleavages (Y-Axis) and noise factors (X-Axis). Higher AA recall is shown in green for pNovo 3 (A), yellow for SMSNet (B), orange for PointNovo (C), and red for Casanovo (D). Lower AA recall is displayed in white.


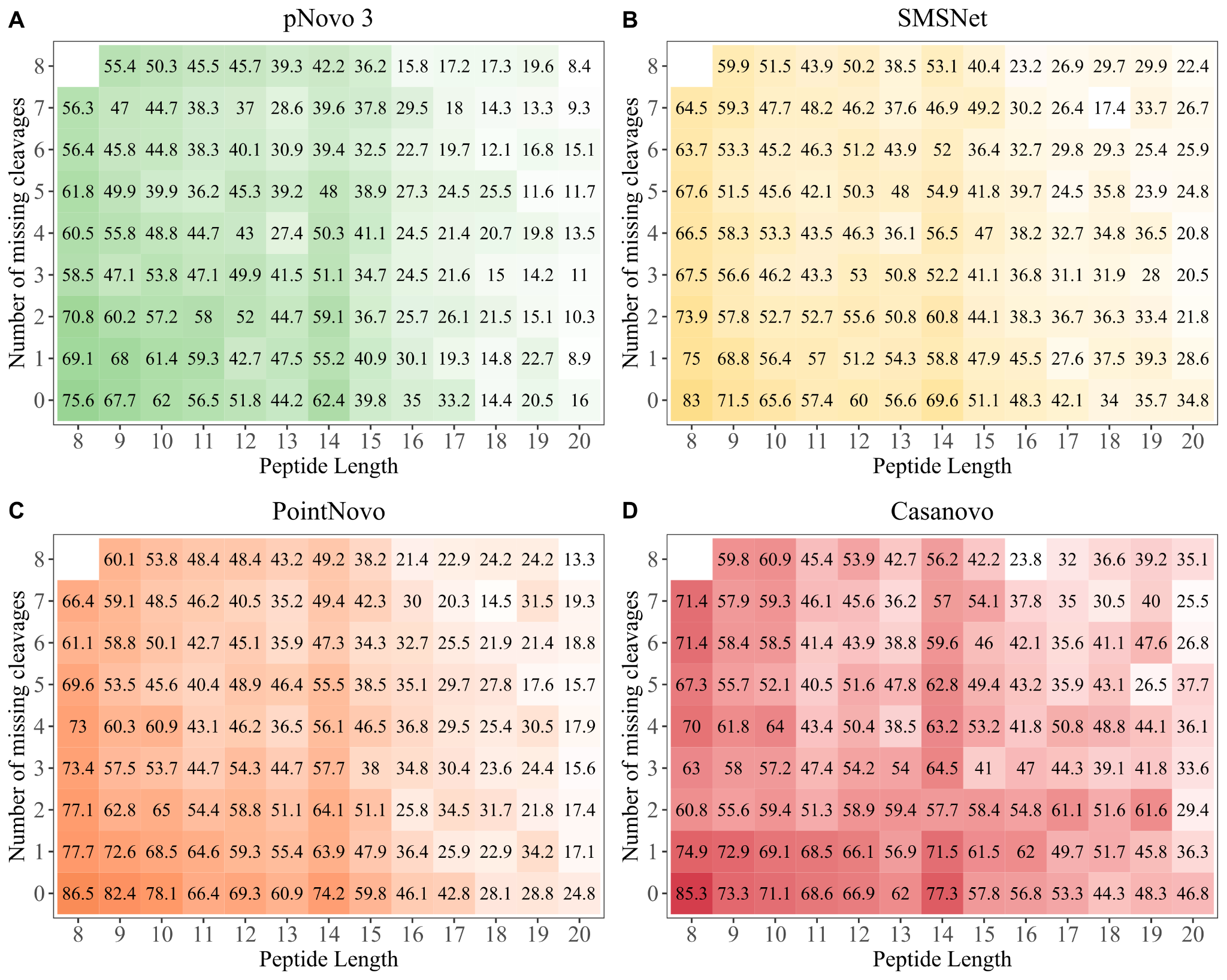


**Supplementary Figure S8** | Heatmap showing amino acid recall for the number of missing cleavages (Y-Axis) and peptide lengths (X-Axis) across all datasets. Higher AA recall is shown in green for pNovo 3 (A), yellow for SMSNet (B), orange for PointNovo (C), and red for Casanovo (D). Lower AA recall is displayed in white.


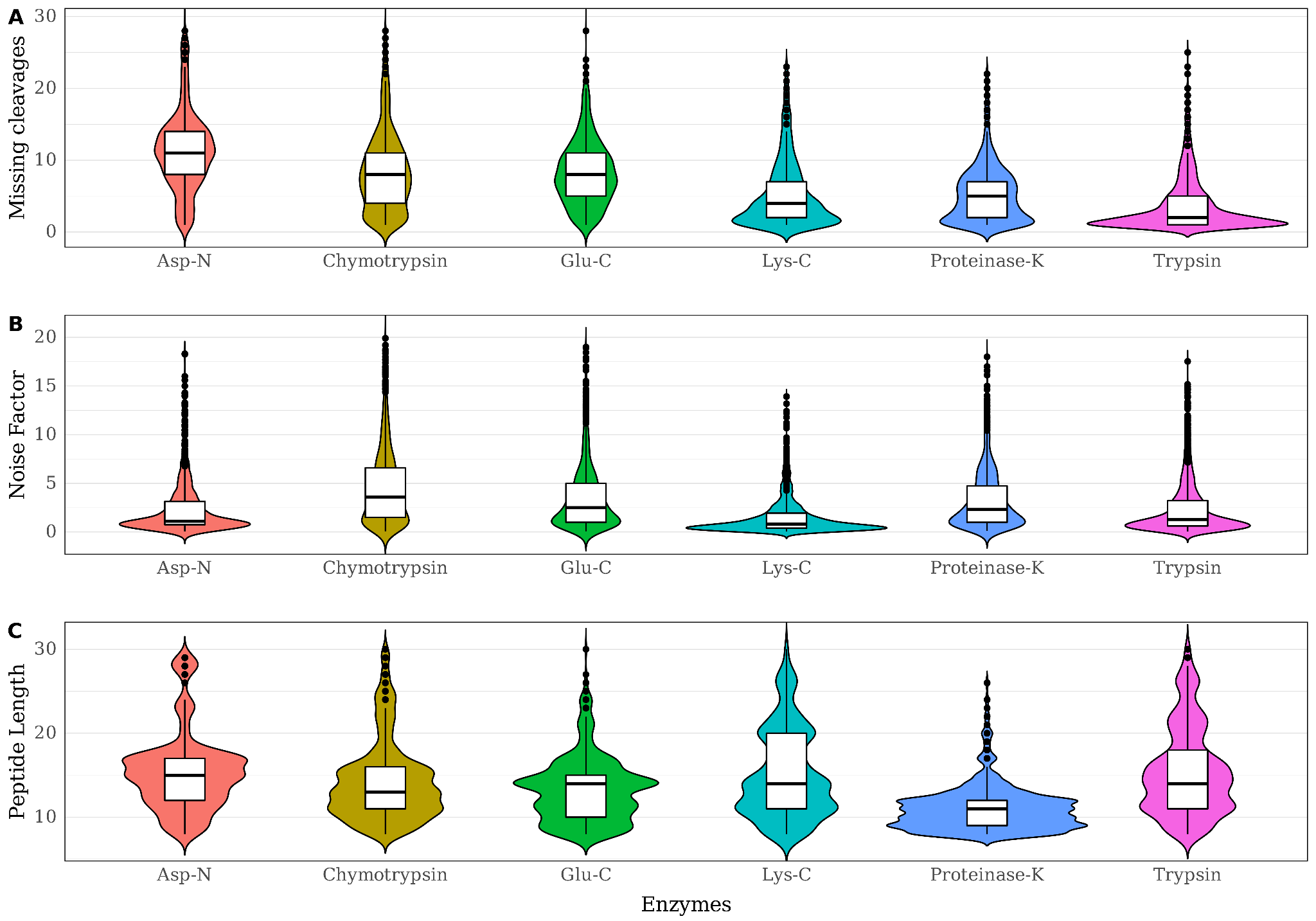


**Supplementary Figure S9** | Violin plots showing the distribution of the number of missing cleavage sites (A), noise factor (B), and length of peptides (C) from different enzymatic datasets of the IgG1-HC. We excluded outlier spectra above a noise of 20 for visualization purposes.


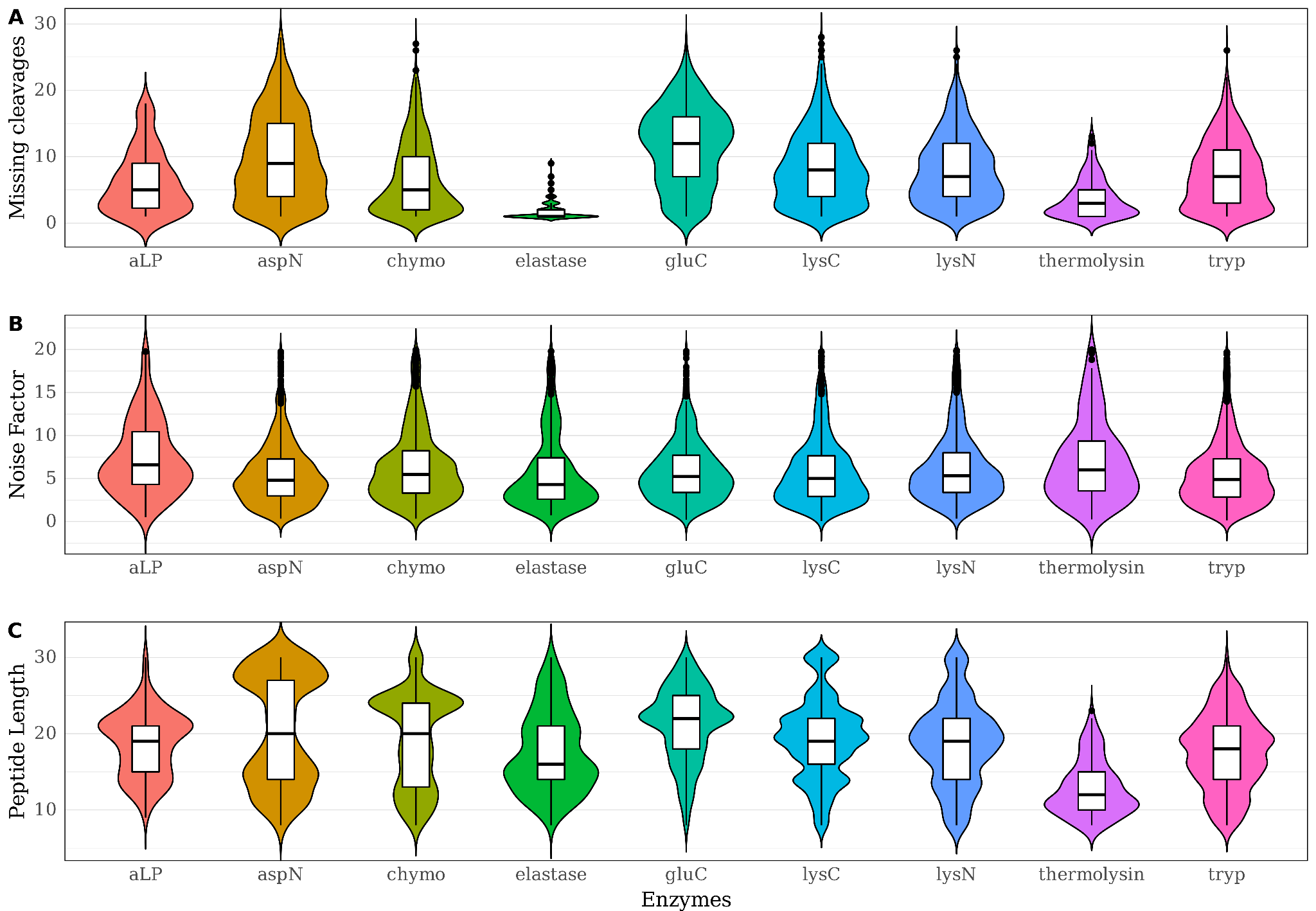


**Supplementary Figure S10** | Violin plots showing the distribution of the number of missing cleavage sites (A), noise factor (B), and length of peptides (C) from different enzymatic datasets of Herceptin. We excluded outlier spectra above a noise of 20 for visualization purposes.

^
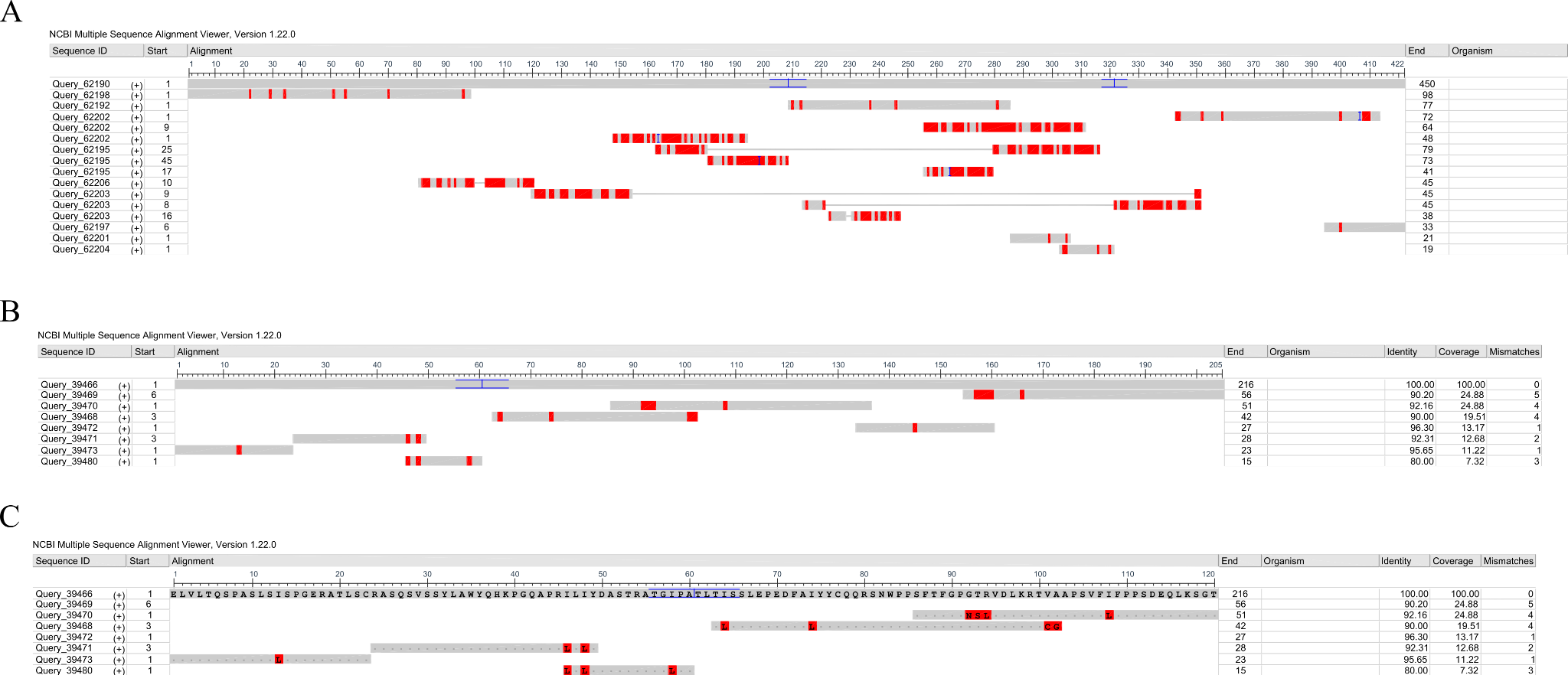
^

**Supplementary Figure S11** | Assembly results for the IgG1-Human. BLAST alignment of the top assembled contigs from PointNovo against the target heavy chain (A). BLAST alignment of the assembled contigs from PointNovo against the target light chain (B). BLAST alignment of the assembled contigs from PointNovo against the variable region of the target light chain (C).


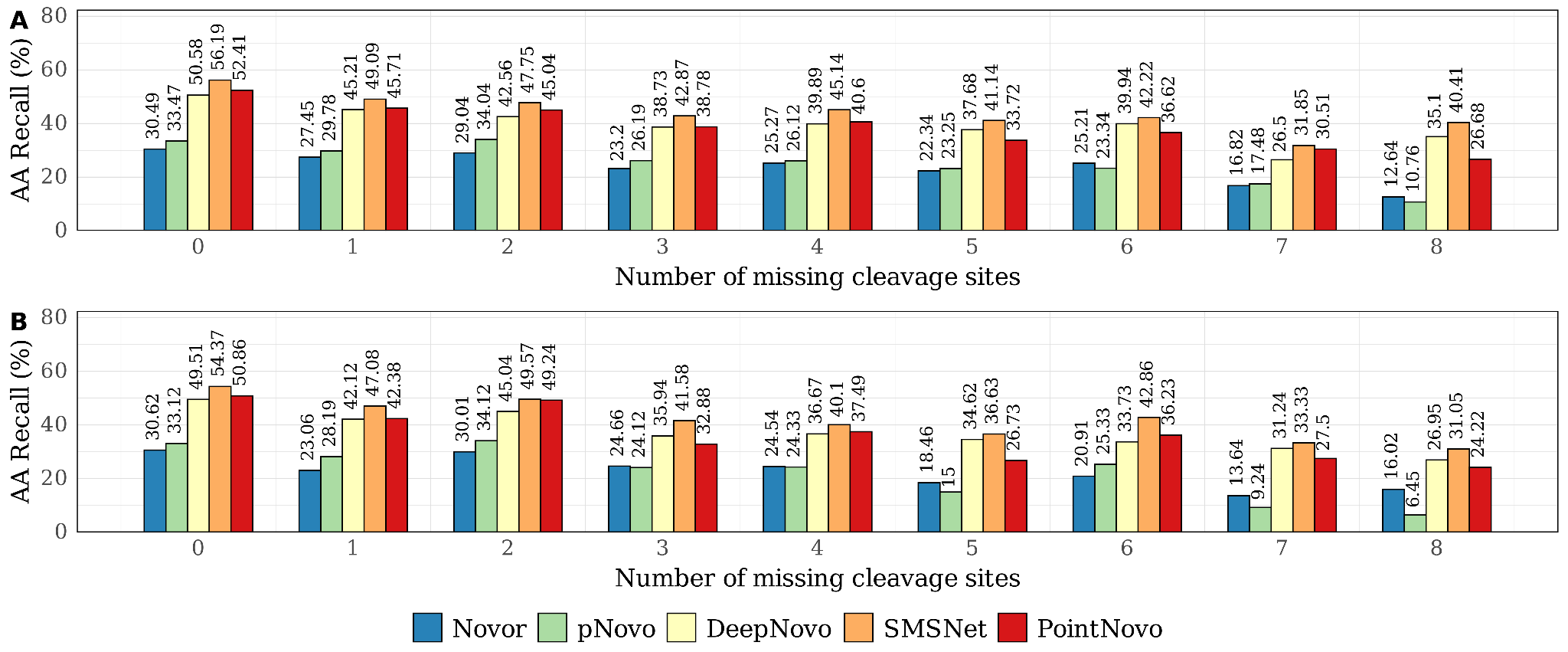


**Supplementary Figure S12** | Total peptide recall of Novor, pNovo 3, DeepNovo, SMSNet and PointNovo across the WIgG1 HC dataset for different number of cleavage sites missing. To evaluate the impact of a-ions, we compared the peptide recall for fragmentation sites with 8 (b and y ions) missing ion types (A) and fragmentation sites with 12 (a, b and y) missing ion types (B).


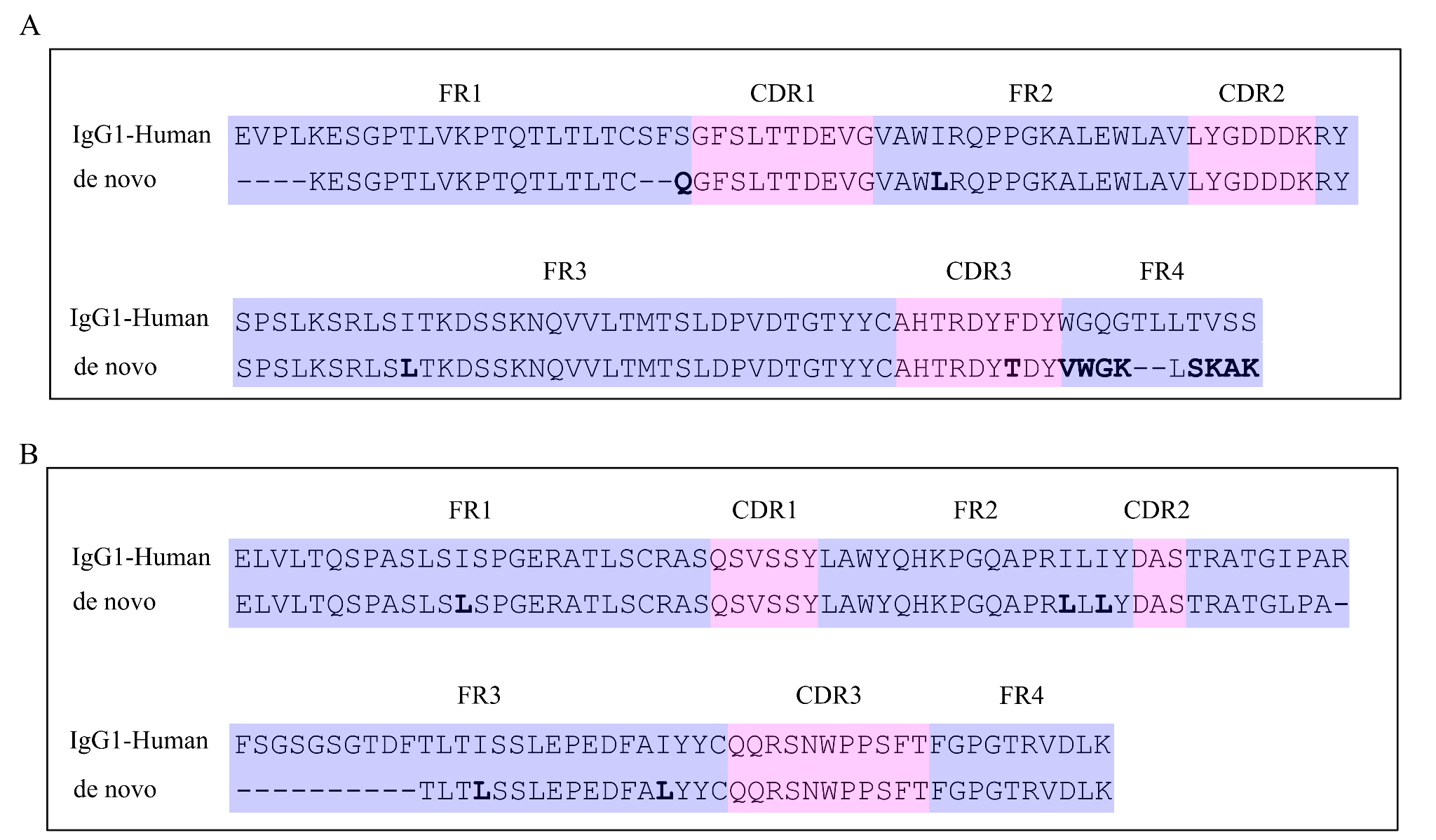


**Supplementary Figure S13** | *De novo* assembly of the variable region of the heavy chain (A) and light chain (B) of IgG1-Human. We used all available enzymes for *de novo* sequencing with PointNovo and assembly with ALPS. We aligned the resulting contigs (5 for Heavy chain and 4 for Light Chain) against the target protein to reconstruct the antibody sequence. CDRs are marked in pink, while framework regions (FRs) are marked in blue. Mismatches are indicated by bold characters. Gaps are indicated by dashes. FRs and CDRs were numbered according to the Natural Antibody database (research.naturalantibody.com/pad).

**Supplementary Table S1** | Incorrect amino acid substitutions made by *de novo* sequencing tools pNovo 3, SMSNet, and PointNovo on datasets of IgG1-Human, WIgG1-Mouse, and Herceptin. Shown is the relative amount of specific amino acid substitutions for sequencing errors, where only 1 AA was incorrectly predicted

| Type of Replacement | pNovo 3 | SMSNet | PointNovo |
| --- | --- | --- | --- |
| N (deam.) ⇔ D | 59.73 % | 41.64 % | 74.51 % |
| N ⇔ D | 0.84 % | 10.18 % | 7.89 % |
| Q (deam.) ⇔ E | 28.11 % | 7.96 % | 8.86 % |
| K ⇔ Q | 0.00 % | 11.09 % | 0.00 % |
| Other | 11.32 % | 29.13 % | 8.74 % |

**Supplementary Table S2** | Error types made by *de novo* sequencing tools pNovo 3, SMSNet, PointNovo, and Casanovo on the datasets of IgG1-Human, WIgG1-Mouse and Herceptin using only peptides without missing cleavages. Shown is the relative amount of 11 different error types for each algorithm.

| Type of Error | pNovo 3 | SMSNet | PointNovo | Casanovo |
| --- | --- | --- | --- | --- |
| Number of total predictions | 2203 | 2203 | 2203 | 2376 |
| Number of total errors | 1589 | 1567 | 1385 | 429 |
| Inversion first 3 AAs | 6.3% | 10% | 7.5% | 12% |
| Inversion last 3 AAs | 2.3% | 4.3% | 4.1% | 10% |
| Inversion first & last 3 AAs | 0.0% | 0.1% | 0.1% | 0.7% |
| 1 AA replaced by 1 AA or 2 AAs | 25% | 11% | 23% | 36% |
| 2 AAs replaced by 2 AAs | 7.2% | 11% | 9.7% | 9.1% |
| 3 AAs replaced by 3 AAs | 5.4% | 5.9% | 7.5% | 4.4% |
| 4 AAs replaced by 4 AAs | 2.0% | 4.0% | 2.9% | 4.0% |
| 5 AAs replaced by 5 AAs | 2.5% | 3.7% | 2.4% | 2.1% |
| 6 AAs replaced by 6 AAs | 4.5% | 1.8% | 4.8% | 1.2% |
| More than 6 AAs wrong | 28% | 30% | 29% | 7.5% |
| Other | 17% | 18% | 8.2% | 12% |

**Supplementary Table S3** | Summary of *de novo* assembly results on heavy chains of three antibody datasets using ALPS (k=7). The top 20 contigs were used to compare the length, coverage, and accuracy of mapped contigs. Mapped contigs must be aligned to the reference protein sequence. The longest contig describes the maximum length of all generated contigs. Sequence coverage was calculated as the percentage of amino acids of the protein sequence that were covered by at least one contig. Accuracy was calculated as the percentage of all protein sequence calls that were correctly labeled.

|  | IgG1 HC (446 AA) | WIgG1 HC (441 AA) | Herceptin HC (449 AA) |
| --- | --- | --- | --- |
| **pNovo 3** | | | |
| Mapped contigs | 15 | 14 | 14 |
| Longest contig (AA) | 34  (7.62%) | 22  (4.99%) | 42  (9.35%) |
| Sequence coverage (%) | 307  (68.83%) | 211  (47.85%) | 314  (69.93%) |
| Sequence accuracy (%) | 262  (85.34%) | 181  (85.78%) | 242  (77.07%) |
| **SMSNet** | | | |
| Mapped contigs | 13 | 9 | 7 |
| Longest contig (AA) | 65  (14.57%) | 71  (16.10%) | 83  (18.49%) |
| Sequence coverage (%) | 391  (87.67%) | 340  (77.10%) | 314  (69.93%) |
| Sequence accuracy (%) | 348  (89.00%) | 299  (87.94%) | 270  (85.99%) |
| **PointNovo** | | | |
| Mapped contigs | 15 | 10 | 6 |
| Longest contig (AA) | 75  (16.81%) | 66  (14.97%) | 98  (21.82%) |
| Sequence coverage (%) | 437  (97.98%) | 370  (83.90%) | 291  (64.81%) |
| Sequence accuracy (%) | 361  (82.61%) | 347  (93.78%) | 269  (92.44%) |
| **Casanovo** |  |  |  |
| Mapped congis | 9 | 7 | 7 |
| Longest contig (AA) | 83  (18.61%) | 83  (18.82%) | 79  (17.59%) |
| Sequence coverage (%) | 418  (93.72%) | 388  (87.98%) | 343  (76.39%) |
| Sequence accuracy (%) | 410  (98.09%) | 368  (94.85%) | 323  (94.17%) |

**Supplementary Table S4** | Sequence coverage and accuracy of the identified CDRs of three antibodies using PointNovo and ALPS.Sequence coverage (Cov.) was calculated as the number and percentage of amino acids of the CDR that were covered by at least one contig. Accuracy (Acc.) was calculated as the percentage of all CDR sequence calls that were labeled correctly. The number in the brackets shows the percentage of correct coverage and accuracy compared to the target sequence. Misidentifications between leucine and isoleucine were not counted as incorrect. CDRs were identified via the Natural Antibody database (research.naturalantibody.com/pad).

|  | CDR1 | | CDR2 | | CDR3 | |
| --- | --- | --- | --- | --- | --- | --- |
| Antibody | Cov. | Acc. | Cov. | Acc. | Cov. | Acc. |
| IgG-1 Human HC | 10 of 10 (100%) | 10 of 10 (100%) | 7 of 7 (100%) | 7 of 7 (100%) | 9 of 9 (100%) | 8 of 9 (88%) |
| IgG-1 Human LC | 6 of 6  (100%) | 6 of 6 (100%) | 3 of 3 (100%) | 3 of 3 (100%) | 9 of 9 (100%) | 9 of 9 (100%) |
| WIgG Mouse HC | 0 of 8  (0%) | 0 of 0  (0%) | 7 of 7 (100%) | 6 of 7 (85%) | 12 of 12 (100%) | 11 of 12 (91%) |
| WIgG Mouse LC | 11 of 11  (100%) | 10 of 11 (91%) | 3 of 3 (100%) | 3 of 3 (100%) | 9 of 9  (100%) | 9 of 9  (100%) |
| Herceptin HC | 8 of 8  (100%) | 8 of 8 (100%) | 8 of 8 (100%) | 7 of 8 (87%) | 9 of 13  (69%) | 2 of 9  (22%) |
| Herceptin LC | 6 of 6  (100%) | 5 of 6  (83%) | 3 of 3  (100%) | 3 of 3 (100%) | 9 of 9 (100%) | 8 of 9  (88.8%) |

**Supplementary Table S5** | Sequence coverage and accuracy of the identified CDRs of three antibodies using Casanovo and ALPS. Sequence coverage (Cov.) was calculated as the number and percentage of amino acids of the CDR that were covered by at least one contig. Accuracy (Acc.) was calculated as the percentage of all CDR sequence calls that were labeled correctly. The number in the brackets shows the percentage of correct coverage and accuracy compared to the target sequence. Misidentifications between leucine and isoleucine were not counted as incorrect. CDRs were identified via the Natural Antibody database (research.naturalantibody.com/pad).

|  | CDR1 | | CDR2 | | CDR3 | |
| --- | --- | --- | --- | --- | --- | --- |
| Antibody | Cov. | Acc. | Cov. | Acc. | Cov. | Acc. |
| IgG-1 Human HC | 8 of 10  (80%) | 8 of 8 (100%) | 7 of 7 (100%) | 7 of 7 (100%) | 9 of 9 (100%) | 8 of 9 (88%) |
| IgG-1 Human LC | 6 of 6  (100%) | 6 of 6 (100%) | 3 of 3 (100%) | 3 of 3 (100%) | 9 of 9 (100%) | 9 of 9 (100%) |
| WIgG Mouse HC | 0 of 8  (0%) | 0 of 0  (0%) | 7 of 7 (100%) | 7 of 7 (100%) | 12 of 12 (100%) | 11 of 12 (91.7%) |
| WIgG Mouse LC | 11 of 11  (100%) | 9 of 11 (81%) | 3 of 3 (100%) | 2 of 3 (66%) | 9 of 9  (100%) | 9 of 9  (100%) |
| Herceptin HC | 8 of 8  (100%) | 8 of 8 (100%) | 8 of 8 (100%) | 8 of 8 (100%) | 11 of 13  (84%) | 10 of 11  (90%) |
| Herceptin LC | 6 of 6  (100%) | 5 of 6  (83.3%) | 3 of 3  (100%) | 3 of 3 (100%) | 9 of 9 (100%) | 9 of 9  (100%) |
